# Short Communication: Some Observations on the Role of Bradykinin in Immunity to *Teladorsagia circumcincta* in Sheep

**DOI:** 10.1155/2012/569287

**Published:** 2012-02-23

**Authors:** Andrew R. Williams

**Affiliations:** School of Animal Biology, The University of Western Australia, Crawley, WA 6009, Australia

## Abstract

Bradykinin is a physiologically active peptide involved in vasodilation and smooth muscle contraction and is previously shown to be increased in gastrointestinal mucus during nematode challenge in sheep. Here, it is shown that bradykinin in the abomasum is positively correlated with both mast cells and globule leukocytes in the abomasum, and that all three of these parameters are negatively associated with numbers of adult *Teladorsagia circumcincta* during the challenge of immune sheep. It is suggested that bradykinin either stimulates the degranulation of mast cells, or is released during this degranulation process, or both. Multiple regression showed that almost 60% of the variation of in adult *T. circumcincta* could be explained by two variables, bradykinin and *T. circumcincta*—specific IgG_1_ in plasma. This provides further evidence that bradykinin may be a mechanism of protective immunity in sheep, although its involvement in asthma and other allergic disorders raises questions about its role in unwanted immunopathology.

## 1. Introduction

Helminth infections invariably invoke Th2-like immune responses in mammalian hosts, characterised by production of IgE, IgG_1_, mucosal mast cells, and eosinophils [1]. In addition, we have recently shown that the peptide bradykinin is significantly increased in abomasal mucus during helminth challenge in immune sheep [2]. Bradykinin is a potent vasodilator that, during inflammation, is cleaved from circulating kininogen and is normally associated with endothelial permeability and nonvascular smooth muscle contraction in conditions such as asthma [3, 4]. It was found that abomasal bradykinin was higher in sheep with lower total worm burdens, suggesting that it may be involved with protective immunity. However, it is not clear what role bradykinin plays and how it interacts with other immune mechanisms. The present communication reports associations between bradykinin and granulocytes and antibodies produced during helminth challenge in sheep.

## 2. Methods

### 2.1. Parasites and Animals

The experimental design of the animal trial is described in detail in Williams et al. [2]. Forty Merino sheep were housed indoors and challenged daily with 500 third-stage larvae each of *Teladorsagia circumcincta *and *Trichostrongylus colubriformis. *The sheep were from a line selectively bred for low faecal worm egg count (FEC) and had been raised on pastures contaminated with helminth larvae and thus had acquired a degree of protective immunity [5]. Total worm burdens were quantified after six weeks of continuous challenge.

### 2.2. Immunological Measurements

IgG_1_ specific to *T. circumcincta *and *T. colubriformis *L_3_ antigen was determined by ELISA four weeks after challenge began and was reported as ELISA units relative to a positive control (high-reading sera). At postmortem, tissue samples were taken from the abomasum and proximal third of the small intestine, fixed in 4% paraformaldehyde and the numbers of mast cells, globule leukocytes, and eosinophils enumerated by standard histological methods and expressed as cells per/mm^2^ of tissue. Mucus was snap-frozen in liquid nitrogen for bradykinin analysis. Bradykinin concentrations were measured in mucus after purification by solid-phase extraction, using a commercial ELISA kit according to the manufacturers instructions. Bradykinin levels were expressed per mg of protein in mucus samples. Further details are provided in Williams et al. [2, 6].

### 2.3. Statistical Analysis

Worm burden data was transformed (log⁡_10_⁡(*N* + 10)) before analysis. Other data was normally distributed and analysed untransformed. Simple and multiple regression was used to determine correlations and relationships between variables, using the STATISTICA program (version 10, StatSoft, Tulsa, OK, USA).

## 3. Results

**Figure 1:**
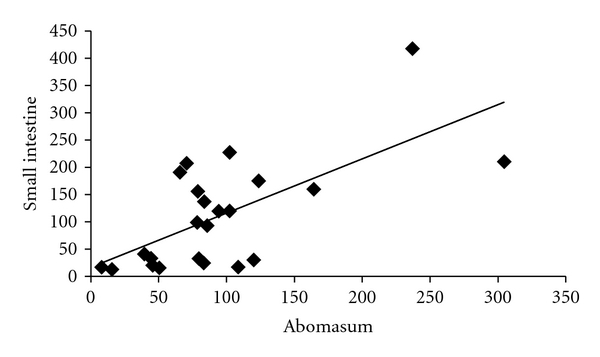
Relationship between concentrations of bradykinin (pg/mg protein) in abomasal mucus and the corresponding concentration in small intestinal mucus. *r* = 0.66, *P* < 0.001.

Concentrations of bradykinin in the abomasum were positively correlated (*r* = 0.66, *P* < 0.001) with concentrations of bradykinin in the small intestine (Figure 1). However, the regression accounted for only 44% of the variation in abomasal bradykinin concentration (Table 1). Similarly, numbers of all three types of cell measured (eosinophils, mast cells, and globule leukocytes) were positively correlated between the two sites of infection (Table 1).

**Figure 2:**
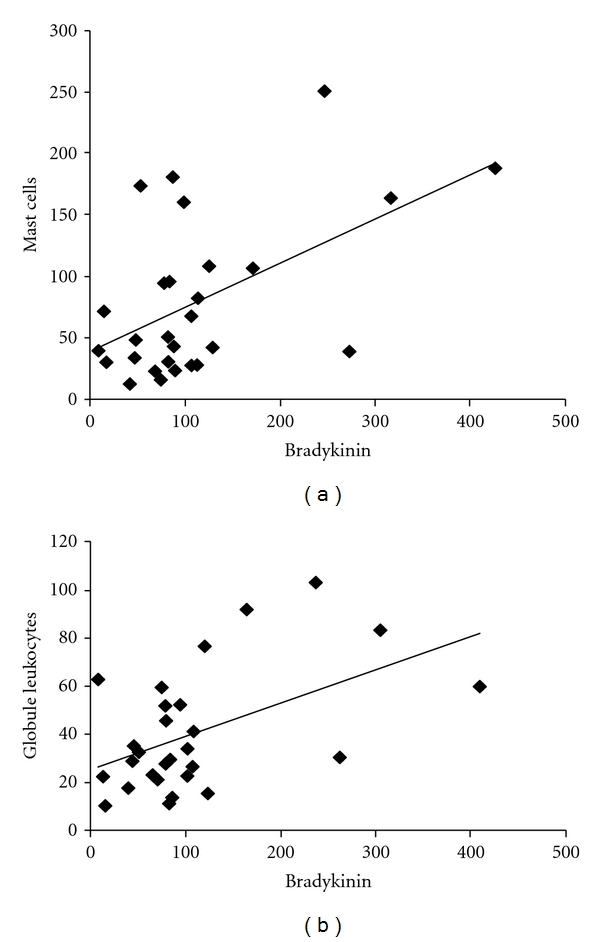
Relationships between the concentration of bradykinin in abomasal mucus and the numbers of mast cells (*r* = −0.53, *P* = 0.003, *n* = 28) and the numbers of globule leukocytes (*r* = −0.5, *P* = 0.006, *n* = 28) in the abomasum.

The relationships between immune mechanisms and parasite burdens were reported in Williams et al. [2]. Briefly, bradykinin in the abomasum was negatively correlated (*r* = −0.62, *P* < 0.001) with adult *T. circumcincta *but not larval stages of *T. circumcincta*. In contrast, there was no relationship between intestinal bradykinin and numbers of *T. colubriformis, *nor was intestinal bradykinin correlated with numbers of granulocytes in the small intestine mucosa. Abomasal bradykinin was positively associated with both mast cells and globule leukocytes in the abomasum (Figure 2). Mast cells and globule leukocytes in the abomasum were also negatively correlated with numbers of adult *T. circumcincta *(Table 2), but multiple regression showed that the same amount of variation in *T. circumcincta *number was explained by bradykinin alone or when added to the model together with mast cells, or globule leukocytes (Table 2). In fact, neither mast cells or globule leukocytes were a significant variable when bradykinin was added to the model, suggesting that it is bradykinin rather than granulocytes which is responsible for the majority of the variation in *T. circumcincta *number.

 IgG_1_ specific for *T. circumcincta *was negatively correlated with adult *T. circumcincta *burdens (*r* = −0.57, *P* < 0.01), but not correlated with bradykinin. When bradykinin and IgG_1_ were included in a multiple regression analysis, they accounted for almost 60% of the variation in total adult *T. circumcincta *burdens, compared to 30% for IgG_1_ alone and 38% for bradykinin alone (Table 2).

## 4. Discussion

Bradykinin is clearly associated with immunity to *T. circumcincta *in sheep. The mechanisms whereby bradykinin production may contribute to nematode expulsion have not been elucidated. However, given bradykinin's role in vasodilation and airway bronchoconstriction in asthma, it may be that it contributes to mucus hypersecretion, plasma leakage into the gut, and increased peristalsis—similar to the role proposed for other inflammatory mediators such as leukotrienes that have been repeatedly been shown to be involved in immunity to gastrointestinal nematodes [2, 7, 8]. A similar relationship between bradykinin and *T. colubriformis *was not observed in this experiment, but that is likely a result of the experimental sheep or being extremely resistant to infection with this parasite, and thus, there was little variation to determine relationships between worm burdens and immune effector mechanisms [2].

Eosinophils, mast cells, and globule leukocytes are invariably increased in the gut mucosa during nematode infection in sheep and thought to be involved in protective immunity [1], and the positive correlations shown here between cell numbers in adjacent segments of the gut indicate that there appears to be a “generalised” nematode immunity, that is, sheep that have a heightened cellular response to one parasite also have this high response against a second parasite species. This suggests that sheep bred to be resistant to nematodes on the basis of exposure to one parasite species may also exhibit increased immunity when challenged with a heterologous species. The positive correlation between bradykinin concentrations in the abomasum and small intestine, also provides evidence for this concept.

The associations between bradykinin and mast cells/globule leukocytes suggest a close relationship between these two mechanisms. Consistent with this, mast-cell-derived heparin has recently been shown to initiate bradykinin formation in mouse experiments, leading to marked vascular permeability [9]. A similar process may operate in sheep, whereby mast cells stimulated to degranulate by nematode antigens lead to bradykinin formation which directly leads to plasma leakage into the gut. This leakage is thought to be an important component of protective immunity to gastrointestinal parasites, perhaps due to plasma leakage allowing antibodies to come into contact with nematodes at the mucosa/lumen interface. Consistent with this hypothesis, regression analysis in the current paper showed that bradykinin and IgG_1_ together accounted for a large proportion of the variation in adult worm number. This could be due to mast cell derived-bradykinin dilating blood vessels surrounding the gut mucosa, allowing IgG to come into direct contact with incoming larvae and facilitating their expulsion. Further experiments will be necessary to more rigorously test this hypothesis. *In vitro *studies would be particularly useful to determine the interplay among antigen-dependent activation of mast cells, bradykinin formation, and subsequent parasite growth inhibition. In addition, the contribution of other mechanisms may be important such as mucosal IgA, which was not measured here and has previously been reported to influence worm length and fecundity [10].

A further aspect to be considered is whether increased levels of bradykinin may have unwanted pathological side effects. It has been noted several times that sheep that are highly resistant to helminth parasites can suffer an increase in diarrhoea during natural grazing, and it has been speculated that this may be due to a hypersensitive mucosal immune response leading to larval rejection whilst also causing unwanted immunopathology. Given bradykinin's role in allergic disorders such as asthma, further studies should investigate this relationship in order to determine whether bradykinin may be suitable as a phenotypic marker for nematode resistance in sheep. However, it has previously been reported with these experimental animals that bradykinin levels were not associated with faecal softening during parasite challenge [2].

In conclusion, bradykinin is associated with the mast-cell response to nematode larval challenge in immune sheep and may function together with well-known immune mechanisms such as IgG_1_ to facilitate nematode expulsion.

## Figures and Tables

**Table 1:** Regression models for the relationship between granulocytes and bradykinin in the abomasum (A) and small intestine (S).

Model	*n*	Equation	*r* ^2^	*P* value
Eosinophils (E)	40	EA = (0.37 ± 0.14)ES + 11.5 ± 14.1	0.14	0.018
Mast cells (MC)	40	MCA = (0.38 ± 0.12)MCS + 34.4 ± 16.8	0.15	0.016
Globule leukocytes (GL)	40	GLA = (0.35 ± 0.08)GLS + 16.7 ± 5.8	0.18	0.013
Bradykinin (BK)	24	BKA = (0.66 ± 0.16)BKS + 45.9 ± 11	0.44	<0.001

**Table 2:** Regression equations for the relationship between log_10_ adult *T. circumcincta *burden (Tcirc) and the concentrations of bradykinin in the abomasum (BK), numbers of mast cells (MC) and globule leukocytes (GL) in the abomasum, and concentrations of *T. circumcincta*-specific IgG_1_ in plasma (IgG).

Model	*N*	*R* ^2^		Equation	*P*-value
BK	28	0.38		Tcirc = (−0.62 ± 0.15)BK + 336 ± 58.3	0.0004
MC	40	0.25		Tcirc = (−0.5 ± 0.17)MC + 3 ± 0.18	0.006
GL	40	0.1		Tcirc = (−0.31 ± 0.15)GL + 3 ± 0.21	0.051
BK + MC	28	0.35	BK	Tcirc = (−0.58 ± 0.13)BK	0.003
			MC	Tcirc = (−0.23 ± 0.09)MC + 2 ± 0.04	0.19
BK + GL	28	0.38	BK	Tcirc = (−0.56 ± 0.18)BK	0.005
			GL	Tcirc = (−0.12 ± 0.18)GL + 3 ± 0.21	0.52
IgG	40	0.3		Tcirc = (−0.56 ± 0.16)IgG + 4 ± 0.41	<0.0001
BK + IgG	28	0.58	BK	Tcirc = (−0.51 ± 0.14)BK	0.001
			IgG	Tcirc = (−0.44 ± 0.14)IgG + 4 ± 0.34	0.004
